# Phylogeography of *Schisandra chinensis* (Magnoliaceae) Reveal Multiple Refugia With Ample Gene Flow in Northeast China

**DOI:** 10.3389/fpls.2019.00199

**Published:** 2019-02-25

**Authors:** Jun-Wei Ye, Ze-Kun Zhang, Hong-Fang Wang, Lei Bao, Jian-Ping Ge

**Affiliations:** ^1^State Key Laboratory of Earth Surface Processes and Resource Ecology and Ministry of Education Key Laboratory for Biodiversity Science and Ecological Engineering, College of Life Sciences, Beijing Normal University, Beijing, China; ^2^Germplasm Bank of Wild Species, Kunming Institute of Botany, Chinese Academy of Sciences, Kunming, China

**Keywords:** climber species, genetic diversity, last glacial maximum, refugia, temperate conifers and broadleaved mixed forests

## Abstract

Temperate conifers and broadleaved mixed forests in northeast China are ideal to investigate the genetic consequences of climate changes during the last glacial maximum (LGM), 29 – 16 kya. As previous studies were focused on tree species with long generation time; here, the evolutionary history of *Schisandra chinensis*, a climber species with a generation time of five years, was investigated using chloroplast DNA (cpDNA), nuclear single copy gene (nSCG), and nuclear single sequence repeats (nSSRs, i.e., microsatellite) markers, along with ecological niche modeling (ENM), which predicted a suitable habitat in Korea Peninsula (KP) during the LGM. Private haplotypes and high genetic diversity of both cpDNA and nSCG were mainly found in KP and Changbai Mt. (CB). Although no significant phylogeographic structure was detected in the cpDNA and nSCG, three nSSRs clusters roughly distributed in west (CB and KP), east (north China), and north (Xiaoxing’an Range, XR) regions were found in Structure analysis. The approximate Bayesian computation analysis showed the west cluster diverged at 35.45 kya, and the other two clusters at 19.85 kya. The genetic diversity calculated for each of the three markers showed no significant correlation with latitude. Genetic differentiation of nSSRs was also not correlated with geographic distance. Migrate analysis estimated extensive gene flow between almost all genetic cluster pairs and BOTTLENECK analysis showed that few populations experienced severe bottlenecks. Overall, results indicate that *S. chinensis* survived the LGM *in situ* in multiple refugia, which likely include two macrorefugia (KP and CB) and two microrefugia (XR and north China). Extensive postglacial gene flow among the three nSSRs clusters led to uniformly distributed genetic diversity and low genetic differentiation.

## Introduction

Quaternary climate changes, especially the last glacial maximum (LGM, 29–16 kya; Clark and Mccabe, 2009), have greatly influenced the distribution of temperate forests in the Northern Hemisphere (Hewitt, 1996, 2000; Qiu et al., 2011). Contractions during glaciations and expansions between or after glaciation periods of species distributions have left imprints on the genetic structure and distribution of genetic diversity and differentiation (Provan and Bennett, 2008; Excoffier et al., 2009; Waters et al., 2013). In northeast China, the temperate conifer and broadleaved mixed forests (hereafter mixed forests) between 40 and 50° N are the contact zone between the southern warm-temperate forests and the northern cool-temperate forests (Supplementary Figure S1; Wu, 1980). Because the mixed forests are sensitive to climate cooling and warming, it is an ideal system to investigate the genetic consequences of the LGM (Wu, 1980; Zhang, 2007).

Vegetation reconstructions using fossil pollen data showed that the mixed forests retreated southward to 25–30° N during the LGM (Harrison et al., 2001; Cao et al., 2015). However, phylogeographic studies provided different scenarios. Two scenarios have been proposed, which are the single refugium scenario and the multiple refugia scenario (Supplementary Figure S1). The single refugium scenario suggests that Changbai Mt. (CB), located at about 40–42° N, is the only macrorefugia for conifer trees within the genus *Abies* (Jiang et al., 2011), the broadleaf tree *Juglans mandshurica* (Bai et al., 2010), and the perennial herb *Buplerum longiradiatum* (Zhao et al., 2013). The multiple refugia scenario suggests the Korea Peninsula (KP) as another macrorefugia in addition to CB (Guo et al., 2014; Wang et al., 2014; Bao et al., 2015; Zeng et al., 2015). Other microrefugia were also detected, with the northernmost ones located at the northern mixed forests margin [Xiaoxing’an Range (XR) and Russian Far East] (Bao et al., 2015; Zeng et al., 2015; Wang S.H. et al., 2016). Most species examined were tree and shrub species. The only climber species that has been studied is *Actinidia arguta*, but its limited genetic variation in chloroplast DNA (cpDNA) hindered the precise discrimination of the two different scenarios (Ye et al., 2018). Thus, whether climber species would follow the single refugium or multiple refugia scenario remains unknown.

Genetic diversity and genetic differentiation distribution patterns in mixed forests are also complex (Ye et al., 2017). Genetic diversity is expected to show significant declines due to genetic drift and bottlenecks during the northward expansion from a single source (such as the CB macrorefugia) (Excoffier et al., 2009; Waters et al., 2013). The reduction of genetic diversity in nuclear microsatellites (nuclear single sequence repeats, nSSRs) of *Acer mono* with increasing latitude agrees with this prediction (Guo et al., 2014; Liu et al., 2014). However, *J. mandshurica* shows an inconsistent pattern. Gradual expansion with gravity-mediated seed dispersal combined with extensive wind-mediated pollen gene flow have prevented the decrease of its genetic diversity in the direction of range expansions (Wang W.T. et al., 2016). In addition, if there was substantial postglacial southward expansion from northern microrefugia (such as XR), the genetic diversity would also become uniformly distributed (Bao et al., 2015; Zeng et al., 2015). Genetic differentiation can also show different patterns. An increased genetic differentiation with increased distance (i.e., isolation by distance, IBD) pattern in re-colonized areas is predicted under rapid range expansion, while ample gene flow among source and colonized populations would erase the IBD pattern (Lafontaine et al., 2013). It should be noticed that the above-mentioned studies were performed on tree species with long generation time. Thus, the distributions of genetic diversity and genetic differentiation in plants with short generation time need additional investigation.

*Schisandra chinensis* (Turcz.) Baill, a common climber species with short generation time (about five years) (Li and Zhang, 2017), was chosen for the present study. Its seeds are dispersed by birds (Yuan, 2007), while its pollen is mainly dispersed by insects and, occasionally, by wind (Ai et al., 2007). The genetic patterns in cpDNA, nuclear single copy gene (nSCG), and nSSRs were combined with ecological niche modeling (ENM) to investigate the evolutionary history of *S. chinensis*. Firstly, genetic structure and potential habitat predictions were used to infer potential refugia. Potential divergence histories were compared and parameters of the most possible scenario were estimated using approximate Bayesian computation (ABC). Then, the correlations between genetic diversity and latitude or IBD were calculated. Historical gene flow was estimated using the maximum-likelihood (ML) algorithm, and whether populations have experienced genetic bottleneck was also estimated.

To further understand the influence of the LGM on mixed forests, the present study aimed to (1) determine whether *S. chinensis* conforms to the single refugium or multiple refugia scenario, and (2) reveal the distribution patterns of genetic diversity and genetic differentiation of *S. chinensis* and their potential causes.

**Table 1:** Details of sample location, sample size, and genetic diversity of chloroplast DNA (cpDNA), nuclear single copy gene (*PEPC*) and nuclear microsatellites (nSSRs) of *Schisandra chinensis*.

Code	Location	Long	Lat	cpDNA			*PEPC*			nSSR						
				*n*_1_	HR_1_	Hap_1_	*n*_2_	HR_2_	Hap_2_	*n*_3_	*A*_O_	*H*_E_	*R*_S_	*P*_AR_	*F*_IS_	*P*_T.P.M._
BS	Baishi, Liaoning, China	124.78	40.94	8	0.607	1, 2, 3	10	0.789	1, 2, 3, 4, 5	24	75	0.76	5.09	0.04	0.09	0.24
BX	Fangzheng, Heilongjiang, China	128.99	45.67	8	0.429	2, 3	10	0.816	1, 2, 3, 4, 5	27	67	0.74	4.89	0.28	−0.05	0.22
CB	Changbai Mt., Shenyang, China	127.89	42.25	8	0.250	1, 2	10	0.816	1, 2, 3, 4, 5, 6	21	81	0.81	5.63	0.18	−0.01	0.51
CH	Chunhua, Shenyang, China	131.15	43.39	8	0.250	1, 3	10	0.842	1, 2, 3, 4, 5, 7	34	98	0.82	5.86	0.49	0.01	0.47
DS	Dasuhe, Liaoning, China	125.09	41.88	7	0.524	1, 2, 3	10	0.784	1, 2, 3, 4, 5	14	62	0.72	4.97	0.17	−0.01	0.25
FY	Muling, Heilongjiang, China	130.82	44.79	8	0.464	2, 3, 4	10	0.742	1, 3, 4, 5	16	68	0.79	5.35	0.12	0.10	0.28
HN	Huangnihe, Shenyang, China	128.00	43.61	8	0.250	1, 2	9	0.752	1, 2, 3, 4, 5	14	46	0.72	4.34	0.08	−0.07	0.01
JW	Gariwangsan, Korea	128.56	37.43	8	0.464	2, 3, 5	8	0.858	1, 3, 4, 5, 8, 9, 10	20	82	0.78	5.62	0.29	0.10	0.26
KD	Kundeqi, Heilong Jiang, China	127.74	48.68	8	0.607	1, 2, 3	7	0.780	1, 2, 3, 4	33	76	0.76	4.83	0.09	0.04	0.06
KY	Kunyu Mt., Shandong, China	121.73	37.26	5	0.000	2	5	0.556	1, 3	5	15	0.40	1.88	0.04	−0.94	0.00
LM	Dongling Mt., Beijing, China	115.44	40.01	8	0.000	2	5	0.844	1, 2, 3, 5, 6	8	42	0.64	4.25	0.00	−0.07	0.37
LS	Liangshui, Heilongjiang, China	128.88	47.18	7	0.000	2	7	0.802	1, 2, 3, 4, 5	7	50	0.75	5.36	0.28	0.01	0.55
LW	Longwan, Shenyang, China	126.47	42.46	8	0.536	1, 2	10	0.774	1, 3, 4, 5	32	89	0.82	5.63	0.10	0.03	0.44
MJ	Maojinba, Hebei, China	118.24	41.50	5	0.000	2	5	0.867	1, 2, 3, 4, 6	6	29	0.54	3.37	0.01	−0.10	0.05
QS	Qian Mt., Liaoning, China	123.12	40.98	8	0.000	2	8	0.817	1, 2, 3, 4, 5, 6	8	39	0.59	3.92	0.16	0.09	0.49
RH	Raohe, Heilongjiang, China	133.72	46.69	8	0.679	1, 2, 3	10	0.821	1, 2, 3, 4, 5, 6	25	83	0.79	5.51	0.26	0.10	0.24
WQ	Wangqing, Shenyang, China	130.18	43.35	8	0.429	2, 3	10	0.816	1, 2, 3, 4, 5	25	88	0.82	5.70	0.21	0.11	0.54
WY	Wuyiling, Heilongjiang, China	129.65	48.73	7	0.286	1, 2	10	0.800	1, 2, 3, 4, 5	21	56	0.70	4.52	0.03	−0.05	0.21
XR	Xianrendong, Liaoning, China	122.96	40.02	6	0.000	2	4	0.250	2, 3	6	38	0.67	4.43	0.09	0.15	0.19
ZY	Mt.Jiri, Korea	127.49	35.29	7	0.524	1, 2, 6	9	0.810	1, 2, 3, 11, 12	9	68	0.76	5.96	0.94	0.11	0.25

*Lat, latitude; Long, longitude; HR, haplotype richness; Hap, haplotypes; A_O_: observed allele number; H_E_: expected heterozygosity; R_S_: allelic richness; P_AR_; private allelic richness; and F_IS_: inbreeding coefficient.*

## Materials and Methods

### Sampling

We collected 355 individuals of *S. chinensis* from 20 populations (Table 1) and four individuals of *Kadsura longipedunculata* were collected as outgroups. There were at least 30 m between any two individuals in each population. Silica gels was used to desiccate and preserve all leaf samples. Voucher specimens were deposited at the Beijing Normal University Herbaria (BNU), Beijing, China.

### Chloroplast and Nuclear DNA Sequence Analyses

#### DNA Extraction and Sequencing

Total genomic DNA was extracted using a Plant Genomic DNA Kit (DP305-03; Tiangen, Beijing, China). We amplified and sequenced four chloroplast gene fragments, namely *matK, ndhA*, *trnL-trnF*, and *trnS-trnG* (Supplementary Table S1) for 148 individuals and the nuclear gene *PEPC* for 167 individuals (Table 1). The PCR amplification was performed as described by Guo et al. (2014). The amplicons were sequenced from both directions at the Beijing Genomics Institute (Beijing, China). All electropherograms of DNA sequences were visually analyzed before they were assembled and read in CodonCode Aligner 3.6.1 (CodonCode Corporation^1^, Centerville, MA, United States).

#### Genetic Diversity and Construction of the Most Parsimonious Network

CodonCode Aligner 3.6.1 with the CLUSTAL module was used to align all sequences. The alignment was verified visually and low-quality sections at the beginning and end of sequences were deleted. The PHASE function in DnaSP 5.10.01 (Rozas et al., 2003) was used to determine heterozygous sequences in the nSCG. Then, DnaSP 5.10.01 was used to determine haplotypes, without considering indels, and to calculate nucleotide diversity (π) and haplotype richness (HR). Permut 1.0^2^ with 1000 permutations was used to compare two forms of genetic differentiation (*G*_ST_ and *N*_ST_) in all populations. The most parsimonious network was inferred using Network 4.6.1.1 (Bandelt et al., 1999). Pearson correlations (“corr.test” function) between HR and latitude were performed in R 3.2.3 (R Core Team, 2013).

### Microsatellite Data Analysis

#### Amplification and Genotyping

Eight nSSRs loci were used to determine the genotypes of all sampled individuals in each population (Supplementary Table S2). The PCR procedures were the same as those for amplifying nuclear and cpDNA sequences, except for the annealing temperature (Supplementary Table S2). Amplicons were loaded onto a 3730XL Automated Genetic Analyzer (Applied Biosystems, Foster City, CA, United States) and scored using GeneMapper 4.0 (Applied Biosystems). To reduce score error, alleles were independently read by two people, and any disputes (<5% of calls) were decided by a third person.

#### Genetic Diversity and Bottleneck

The neutral test in Lositan (Antao et al., 2008) indicated that no loci violated the neutral hypothesis; thus, all eight loci were used for further analyses. For each locus and population, standard genetic diversity statistics were calculated. The deviation of the fixation index (*F*_IS_) from zero was used to test the deviation from Hardy–Weinberg equilibrium in each population. Genotypic disequilibrium was tested for all loci pairs in all populations by randomization, and the obtained *P*-values (=0.05) were adjusted using the Bonferroni correction. All the above calculations were performed in Fstat 2.9.3 (Goudet, 2001). Allele richness (*R*_S_) and private allele richness (*P*_AR_) in all populations were calculated in hp-rare 1.0 (Kalinowski, 2005) using rarefaction with a sample number of 10. Pearson correlations (“corr.test” function) between nSSRs genetic diversity (*R*_S_, *P*_AR_, and *H*_*e*_, expected heterozygosity) and latitude were performed in R 3.2.3. Severe population size decreases were detected in BOTTLENECK (Cornuet and Luikart, 1996), applying the two-phase mutation (TPM) with 70% stepwise mutation model (SMM) and 30% multistep mutations.

#### Population Structure

The genetic differentiation index *F*_ST_ (Weir and Cockerham, 1984) was determined for the eight loci using Fstat 2.9.3. Genetic distance, Nei’s *Da*, was used to create a neighbor-joining (NJ) tree using Poptree2 (Takezaki et al., 2010) with 1000 bootstrap pseudoreplicates. Structure 2.3.4 (Falush et al., 2007) was used to detect potential population structure without using population location as prior information. Ten independent runs were performed for each *K* (*K* = 1–20) with 1 × 10^5^ initial burn-in, followed by 1 × 10^6^ Markov Chain Monte Carlo (MCMC) steps using an admixture model with correlated allele frequencies. Potential clusters (*K*) were determined by *LnP(D)*, the change in log-likelihood of the data for each run (Pritchard et al., 2000), and by Δ*K*, the second-order rate of change of *LnP(D)* between successive *K* values (Evanno et al., 2005). Isolation by distance (Wright, 1943) was evaluated according to Rousset (1996) using a Mantel test between genetic differentiation in terms of *F*_ST_/(1 – *F*_ST_) and the natural logarithm of geographic distance. The Mantel test was performed with 9999 permutations in GenAlEx 6.5 (Peakall and Smouse, 2012).

#### Population Divergence History and Gene Flow

To detect population divergence history, all populations were assigned to one of the three gene pools corresponding to its largest proportion of ancestry; population LW (Table 1) was the exception as its proportion of ancestry was approximately identical for the three clusters (see section “Results”). Population divergence history was modeled using the ABC procedure (Beaumont et al., 2002) and performed in DIYABC 2.0 (Cornuet et al., 2014). Three possible divergence history scenarios were compared among the three genetic clusters (see section “Results”; Supplementary Figure S2 and Supplementary Table S3). The simulations were summarized based on the following statistics: mean number of alleles, mean genetic diversity and *F*_ST_ for each lineage, mean classification index, and shared allele distance between pairs of lineages. The simulations were repeated 3,000,000 times and, after logit transformation, local linear regression was applied to choose the 1% simulated data sets that were closest to the observation. Using these data sets, we compared the posterior probability (PP) of the three scenarios and estimated the parameters of the most possible scenario.

The historical gene flow among the nSSRs clusters was estimated using the ML algorithm in Migrate 3.6.8 (Beerli, 2006). We estimated θ = 4*N*μ (*N*, the effective population size; *μ*, the mutation rate per locus per generation) and *M* = *m*/*μ* (*m*, the migration rate per generation) to calculate the effective number of migrants (*Nm*). We used 10 short chains (10,000 trees), three long chains (100,000 trees), 20 genealogies, and burn-in of 10,000 initial trees. The starting values for θ and *M* were estimated from *F*_ST_ values. All individuals were included and the analyses were run three times, independently.

### Ecological Niche Modeling

The maximum entropy modeling technique (MAXENT) (Phillips et al., 2006) was utilized to predict the current potential distribution of *S. chinensis* as well as those during the LGM and last interglacial (LIG). Fifty-five presence records were obtained from this study (20 sample sites) and from the Global Biodiversity Information Facility^3^ (35 occurrence locations). Seven climate variables with low correlation (<0.8) were used to model the niche (Supplementary Table S4) (Hijmans et al., 2005). Model validation was performed 10 times independently, with 20% randomly chosen data. The accuracy of model predictions was evaluated based on the area under the receiver operating characteristic (ROC) curve (AUC) (Fawcett, 2006).

The established model was projected to both MIROC 3.2 ^4^ and CCSM4 (Shields et al., 2012) models to predict potential distributions during LGM and LIG distribution. All climate layers were prepared using a 2.5 arc-minute resolution. The paleocoastlines during the LGM were estimated assuming that the sea level was 130 m lower than the current sea level.

## Results

### cpDNA and nSCG Sequences

**FIGURE 1:**
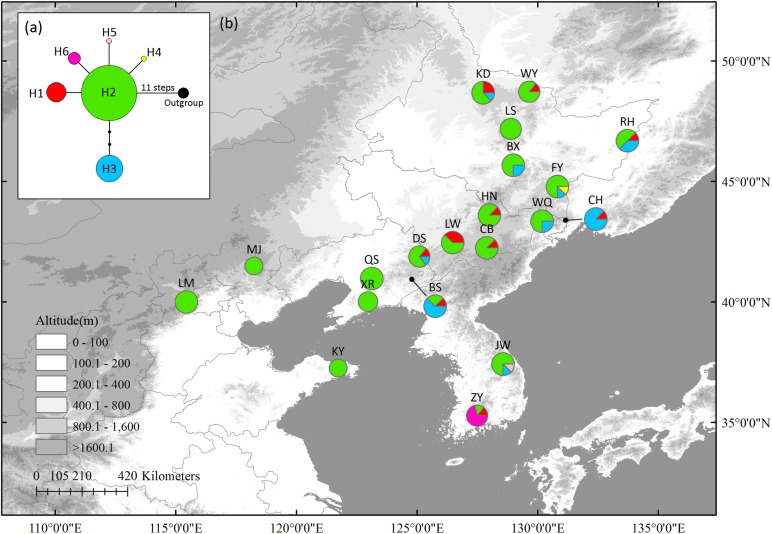
The most parsimonious network **(a)** and geographic distribution **(b)** of haplotypes based on four *Schisandra chinensis* chloroplast fragment, *matK, ndhA*, *trnL-trnF* and *trnS-trnG*. Outgroups represents *Kadsura longipedunculata*. Circle sizes are proportional to sample size of each population.

In cpDNA, seven variable sites, including two singleton variable sites and five parsimony informative sites, were detected (Supplementary Table S5). Six haplotypes were identified with a total alignment length of 3,507 bp (Supplementary Table S5). Haplotype 2 (H2), located in the center of the network, was likely the ancestral haplotype. It was also the most abundant haplotype, being distributed in almost all populations. Only three populations had private haplotypes: FY (H4), in northernmost Changbai Mt., and ZY (H6) and JW (H5) in Korea Peninsula. These three populations also had high HR (Table 1); other populations with high HR were mostly distributed in Changbai Mt. (DS, BS, and LW) and in the northern mixed forests margin (KD and RH) (Figure 1).

**FIGURE 2:**
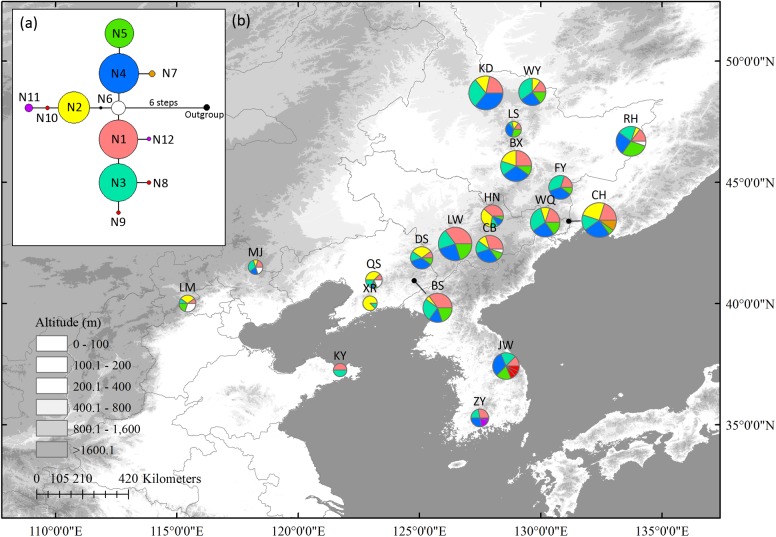
The most parsimonious network **(a)** and geographic distribution **(b)** of haplotypes based on one *Schisandra chinensis* nuclear single copy gene, *PEPC*. Outgroups represents *K. longipedunculata*. Circle sizes are proportional to sample size of each population.

In the nSCG, twelve variable sites, including three singleton variable sites and nine parsimony informative sites, were detected. Twelve haplotypes were identified with a total alignment length of 809 bp (Supplementary Table S6). Because nuclear haplotype 6 (N6) was located in the center of the network, it was likely the ancestral haplotype. It was distributed in north China (LM, QS, and CB) and Changbai Mt. (CB). Populations ZY (N11 and N12), JW (N8 to N10), and CH (N7) in northernmost Changbai Mt. had private haplotypes. Private haplotypes in Korea Peninsula (N8, N9, and N10 in JW, N11 and N12 in ZY) populations were not closely linked (Figure 2). All populations except XR (HR = 0.250) had high HR ranging from 0.556 to 0.867 (Table 1).

Neither significant phylogeographic structure (*N*_ST_ = 0.323 and *G*_ST_ = 0.311 in cpDNA and *N*_ST_ = 0.100 and *G*_ST_ = 0.065 in nSCG, *P* > 0.05) nor correlations between HR and latitude (*r* = 0.21, *P* = 0.38 in cpDNA and *r* = 0.21, *P* = 0.37 in nSCG) were found. All the newly obtained sequences were uploaded to GenBank (Accessions MH580160–MH580199).

### Nuclear Microsatellites

**FIGURE 3:**
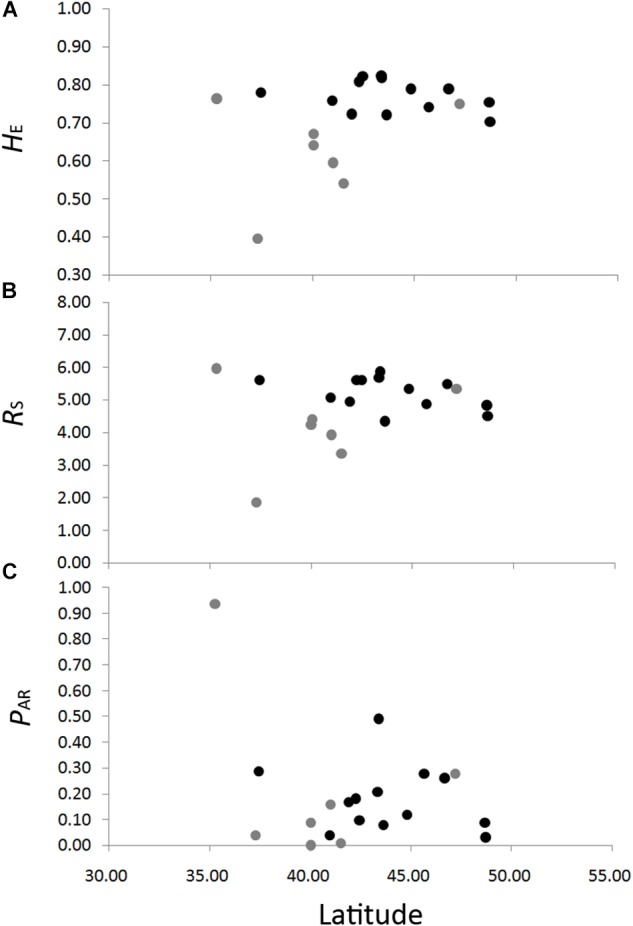
Correlations between genetic diversity, *H*_E_, expected heterozygosity **(A)**; *R*_S_, allelic richness **(B)**; and *P*_AR_, private allelic richness **(C)** in eight nuclear microsatellites, with latitude in *Schisandra chinensis*. Light gray dots represent population with sample size lower than ten.

**FIGURE 4:**
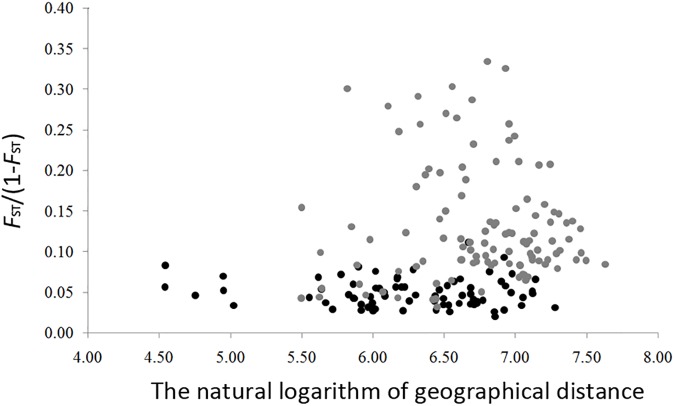
Isolation by distance in *Schisandra chinensis*. Pairwised genetic distance (as measured by *F*_ST_/(1 – *F*_ST_)) is regressed onto the natural logarithm of pairwised geographic distance between all populations. Light gray dots represent population with sample size lower than 10.

The genetic diversity of the eight nSSRs loci and 20 populations are shown in Table 1 and Supplementary Table S7. For each population, *F*_IS_ showed no significant deviation from zero (Table 1), suggesting no violations of the Hardy–Weinberg equilibrium assumptions. No significant genotypic disequilibrium was observed among the 28 loci pairs in any population. No significant correlations between genetic diversity and latitude were found when all populations were considered (*H*_E_, *r* = 0.35, *P* = 0.13; *R*_S_, *r* = 0.20, *P* = 0.40; *P*_AR_, *r* = –0.30, *P* = 0.19) or when populations with sample size < 10 were excluded (*H*_E_, *r* = –0.30, *P* = 0.32; *R*_S_, *r* = –0.45, *P* = 0.12; *P*_AR_, *r* = –0.23, *P* = 0.45) (Figure 3). An IBD pattern was not detected (*r* = 0.22, *P* = 0.06; Figure 4). Two populations (KY and MJ) with limited sample size and population HN experienced bottlenecks (Table 1).

**FIGURE 5:**
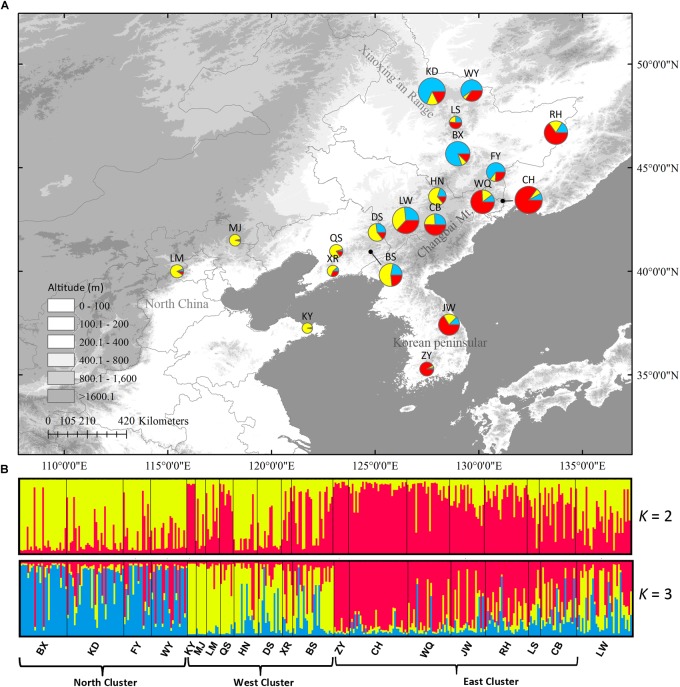
**(A)** Color-coded grouping of the 20 *Schisandra chinensis* populations according to the most likely *K* = 3 in Structure analysis. **(B)** Histogram of the Structure assignment test for all populations at the likely *K* = 2 and 3. Three genetic clusters (west, north and east) were also shown. Circle sizes are proportional to sample size of each population.

In Structure analysis, Δ*K* was high for *K* = 2 or *K* = 3 (Supplementary Figure S3), indicating that populations can be clustered into two or three groups. However, *LnP(D)* and Δ*K* were both higher at *K* = 3 than at *K* = 2, indicating that three clusters were more likely than two clusters. The three clusters were roughly distributed in the east, west and north regions (Figure 5). No population structure was detected using the NJ tree (Supplementary Figure S4).

In the DIYABC analysis, the most possible scenario was that the west cluster diverged before the other two clusters (Supplementary Figure S5, PP = 0.90). The population divergence times between west and north clusters (*t*_2_) and among all three clusters (*t*_1_) were estimated as 3.97 × 10^3^ generations with a 95% highest-probability-density interval (HPD) of 0.79 × 10^3^ – 1.20 × 10^3^) and 7.09 × 10^3^ generations (95% HPD: 1.64 × 10^3^ – 12.40 × 10^3^), respectively. The generation time of *S. chinensis* is assumed to be five years. Therefore, the absolute *t*_2_ was 19.85 kya (95% HPD: 3.95 – 60.00 kya) and the absolute *t*_1_ was 35.45 kya (95% HPD: 8.20 – 62.00 kya). The mutation rate was estimated as 3.91 × 10^−5^/locus/generation. The estimated effective population size in west, north, and east clusters were 4.31 × 10^4^, 8.18 × 10^4^, and 7.13 × 10^5^, respectively (Table 2, Supplementary Figure S5). In Migrate analysis, almost all the estimated gene flows (4*Nm*) were very high (Table 3).

### Ecological Niche Modeling

**FIGURE 6:**
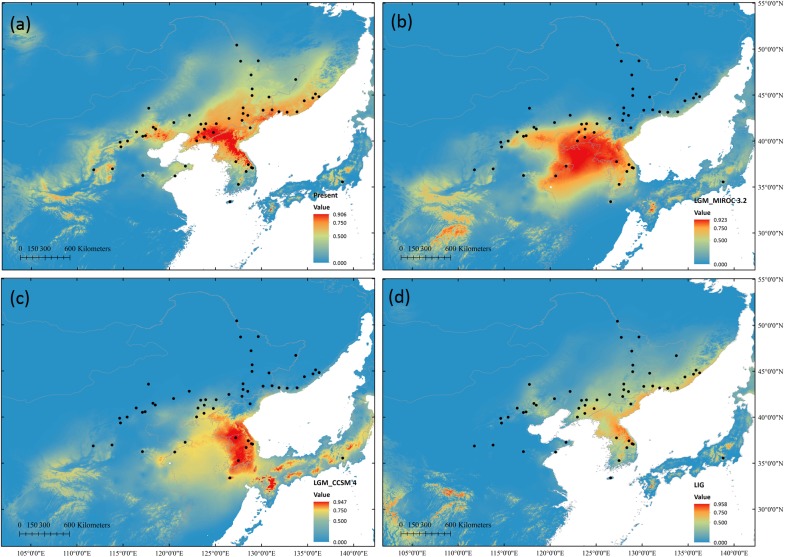
Potential species distribution of *S. chinensis* based on ENM at present **(a)**, during the last glacial maximum using the MIROC 3.2 model **(b)** and in the CCSM4 model **(c)**, and during the last interglacial **(d)**.

High ROC values (0.958 ± 0.012) indicated good accuracy of model predictions. During LGM, both models predicted range contractions. The CCSM4 predicted that the most suitable habitat was located at KP, while MIROC 3.2 indicated the Yellow Sea land bridge and adjacent regions and KP as the most suitable locations. During the LIG, suitable habitats were predicted to be more limited than that at present (Figure 6).

**Table 2:** Posterior median estimation and 95% highest posterior density interval (HPDI) for demographic parameters in the seventh divergence scenario of *S. chinensis* in DIYABC.

	*N*_1_ ( × 10^4^)	*N*_2_ ( × 10^4^)	*N*_3_ ( × 10^5^)	*t*_2_( × 10^3^)	*t*_1_ ( × 10^3^)	*μ* ( × 10^−5^)	*P*
Median	4.31	8.57	7.13	3.97	7.09	3.19	0.42
q(0.05)	1.07	2.64	2.29	0.79	1.64	0.99	0.09
q(0.95)	8.85	16.40	11.30	12.00	12.40	9.99	0.86

*N_1_, N_2_, N_3_: The current population size of east cluster, north cluster, west cluster and ancestral populations at t_2_, respectively; t: divergence time; μ: mutation rate (per generation per locus); P, the proportion of multiple step mutations in the generalized stepwise model, GSM.*

## Discussion

### *In situ* Glacial Survival in Multiple Refugia

Previous phylogeographic studies in mixed forests provide limited information for climber species (Ye et al., 2017). Here, we investigated the evolutionary history of *S. chinensis*, a common climber species in mixed forests, in detail based on multiple genetic markers and potential habitat predictions. Contrary to the southern contraction of mixed forests during the LGM inferred by vegetation reconstructions (Harrison et al., 2001; Cao et al., 2015), the current investigation clearly showed that *S. chinensis* survived the unsuitable climate during the LGM *in situ* in multiple refugia, with the northernmost contacting the northern mixed forests margin.

**Table 3:** Estimates of gene flow (4*Nm*) among three groups of *S. chinensis* based on nuclear microsatellite structure division.

Cluster, *i*	Gene flow (95% HPD)		
	East → *i*	North → *i*	West → *i*
East		59.20 (55.38–63.28)	56.00 (52.33–59.93)
North	58.95 (55.81–62.31)		28.69 (26.90–30.60)
West	4.26 (3.57–5.07)	113.68 (104.26–124.20)	

Low genetic differentiation (*G*_ST_ = 0.311 in cpDNA, *G*_ST_ = 0.065 in nSCG, and *F*_ST_ = 0.130 in nSSRs) was revealed by the different genetic markers and no phylogeographic structure was revealed in either cpDNA and nSCG and in the NJ tree of nSSRs. However, nSSRs Structure analysis indicated that populations could be divided into three clusters (west, north, and east) roughly corresponding to their geographic ranges. The DIYABC analysis showed the west cluster diverged before the other two clusters (35.45 kya) and before the LGM, and persisted through the LGM. The divergence time between the north and east clusters (19.85 kya) indicated their divergence was likely triggered by LGM and these two clusters have persisted *in situ* since the LGM. Thus, the three *S. chinensis* clusters probably had multiple LGM refugia.

The macrorefugia of *S. chinensis* can be inferred through potential habitat prediction. The Korea Peninsula was suggested as one of the macrorefugia for this species, as it provided a suitable habitat during the LGM in both climate models in the ENM. Further, KP populations showed high proportion of private alleles in all three markers indicating long-term persistence. Although the ENM failed to detect a potential suitable habitat in CB during the LGM, populations in CB showed high proportion of private alleles and high genetic diversity in cpDNA and nSCG data. Based on these data and on previous phylogeographic studies (Ye et al., 2017), CB might be another macrorefugia. The Changbai Mountains harbor several forest types, and glacial advances in late Pleistocene only took place at elevations above 2000 m above sea level (Zhang et al., 2008). Thus, low elevation regions with varied vegetation types could serve as a refuge for *S. chinensis*. This hypothesis also explains the existence of the west cluster, which is mainly distributed in KP and CB, and has persisted for longer and much higher effective population size than other two clusters as inferred by the DIYABC analysis.

Potential microrefugia cannot be effectively detected by ENM, because it uses spatially and temporally smoothed climate data, thus being unable to capture climatic variance and the effects of topography on microclimate (Gavin et al., 2014). As proposed by Zeng et al. (2015), our nSSRs also revealed the existence of northern microrefugia. The north nSSRs cluster was mostly distributed in the XR and it diverged during the LGM (19.85 kya) indicating that XR, at the northern mixed forests margin, might be a microrefugium. Moreover, the nSSRs cluster distributed in the east indicated another possible refugium in north China. The ancestral nSCG haplotype N6 distributed mainly in north China also supports the existence of a refugium in this region. Thus, contrasting to the mixed forests’ retreat to 30° N proposed by vegetation reconstructions using fossil pollen data (Harrison et al., 2001; Cao et al., 2015), *S. chinensis* is proposed to have survived the LGM *in situ* in multiple refugia, i.e., in KP, CB, XR, and north China.

Our study on *S. chinensis* provides information on the evolution of another climber species in mixed forests. Ye et al. (2018) suggested repeated expansions and fragmentations in *A. arguta* linked to Pleistocene climate changes have shaped its genetic structure across its distribution from northeast China to subtropical China, although limited cpDNA variation was found. The present study on *S. chinensis* using multiple datasets provides a more detailed evolutionary history of a climber species and a substantial complement to previous mixed forests studies. A brief evolutionary history of mixed forests can be thus be inferred. The mixed forests have survived *in situ* during the LGM using KP and CB as the most important macrorefugia (Jiang et al., 2011; Guo et al., 2014; Wang et al., 2014; Zeng et al., 2015) and microrefugia located in other southern or northern regions were also used. Southern microrefugia can be found in north China (Wang S.H. et al., 2016; Wang W.T. et al., 2016), Shandong Province (Ye et al., 2018), and northern microrefugia can be found in the northern mixed forests margin, such as XR (Bao et al., 2015; Wang S.H. et al., 2016) or the Russian Far East (Zeng et al., 2015).

### Uniformly Distributed Genetic Diversity and Genetic Differentiation

Assuming that the two southern macrorefugia (KP and CB) as sources for northward range expansion, *S. chinensis* is certainly expected to show significant genetic changes along latitude (Excoffier et al., 2009; Waters et al., 2013). However, no significant decreases in genetic diversity, allelic richness, or allelic privacy in organelle and nuclear markers were detected with latitude increase. The same genetic diversity distribution pattern was revealed for *Pinus koraiensis* (Bao et al., 2015). The ABC procedure estimated significant gene flow between southern macrorefugia (KP or CB) and the northern microrefugium XR in *P. koraiensis*. Bao et al. (2015) suggested that this ample gene flow resulted in uniformly distributed genetic diversity and shared dominant haplotypes. In *Quercus mongolica*, Zeng et al. (2015) suggested that if northern microrefugia (such as the Russian Far East) contributed little to post-glacial expansions, the genetic diversity of this species would also show a significant decline. In agreement with that found for *P. koraiensis* (Bao et al., 2015), the present study revealed a northern macrorefugium for *S. chinensis* at XR, and Migrate estimations showed extensive gene flow among the three Structure clusters, the greatest being found from the north to the west cluster (4*Nm*_north→west_ = 113.68, Table 3). Thus, after the *in situ* LGM persistence of the different *S. chinensis* nSSRs clusters, postglacial seed movement by birds (Yuan, 2007), pollen movement by wind (Ai et al., 2007), and the species short generation time led to ample gene flow among the different clusters. Most of the genetic diversity present in macrorefugia (CB and KP) or in the microrefugium (XR) has been preserved in populations in the contact zone among different refugia, and private cpDNA or nSCG haplotypes can only be found in a few *S. chinensis* populations. Therefore, no significant negative correlations with latitude were found for genetic diversity indices using the three independent genetic markers. The homogenizing gene flow among all populations also resulted in the absence of IBD.

## Conclusion

A detailed evolutionary history was revealed for *S. chinensis* using multiple genetic markers and potential habitat predictions. Divergence times among the three nSSRs clusters showed that *S. chinensis* persisted *in situ* through and since the LGM. Two macrorefugia, at KP and CB, were revealed by ENM and high allelic privacy and genetic diversity distributions. Haplotype and nSSRs cluster distributions indicated two possible microrefugia located at XR and north China. Extensive gene flow among clusters resulted in the uniform distribution of genetic diversity and in the absence of an IBD pattern. Short generation time, and seed and pollen movement have facilitated this extensive gene flow. The present study using a common climber species with short generation time is an important complement of previous studies that mainly used tree species with long generation times.

## Data Availability

DNA sequences: deposited in Genbank under accessions MH580160–MH580199. All the chloroplast and nuclear sequences were deposited in TreeBASE (https://www.treebase.org/treebase-web/home.html) under submission number 23007 (http://purl.org/phylo/treebase/phylows/study/TB2:S23007).

## Author Contributions

H-FW, LB, and J-PG conceived the study. Z-KZ contributed to the sampling. Z-KZ and J-WY collected and analyzed the data. J-WY, LB, and H-FW wrote the manuscript. All authors read and approved the final manuscript.

## Conflict of Interest Statement

The authors declare that the research was conducted in the absence of any commercial or financial relationships that could be construed as a potential conflict of interest.

## Footnotes

1 http://www.codoncode.com/aligner/

2 http://www.pierroton.inra.fr/genetics/labo/Software/Permut/

3 http://data.gbif.org

4 http://pmip2.lsce.ipsl.fr/

## Supplementary Material

FIGURE S1 Location of the macrorefugia and microrefugia in the temperate conifers and broadleaved mixed forests inferred by Ye et al. (2017). The blue region indicates the range of the mixed forests following Wu (1980). The 35 occurrence records (black dots) obtained from the Global Biodiversity Information Facility database and from the 20 sampled populations (red dots) are shown.

FIGURE S2 Illustration of the three scenarios **(a–c)** proposed for the divergence history of the three clusters of *Schisandra chinensis* obtained in DIYABC using eight nuclear microsatellites. *N*_1_, *N*_2_, and *N*_3_ represent the effective population sizes of the east, north, and west genetic clusters inferred by Bayesian clustering. Posterior probability (PP) of the different scenarios is shown. Divergence times for the depicted events are labeled as *t*_1_ and *t*_2_.

FIGURE S3 Δ*K* and Ln*P*(*D*) obtained in the Structure analysis of the 20 *Schisandra chinensis* populations conducted for predefined group numbers (*K* = 1–10). The standard deviations of Ln*P*(*D*) obtained from 10 independent runs for each predefined group size are also shown.

FIGURE S4 Neighbor-joining tree of *Schisandra chinensis* populations using Nei’s *D_a_* as the genetic distance of nSSRs. Bootstrap values above 70% are presented above nodes.

FIGURE S5 Prior (red line) and posterior (green line) distribution of *P*, the proportion of multiple step mutations in the generalized stepwise model **(a)**, *t*_2_, divergence time between west and north Structure clusters **(b)**, *t*_1_, divergence time of all three Structure clusters **(c)**, *μ*, mutation rate **(d)**, and effective population sizes of west **(e)**, north **(f)**, and east **(g)** Structure clusters.

TABLE S1 Primers used for chloroplast DNA and nuclear single copy gene sequencings in *Schisandra chinensis*. An annealing temperature of 54 °C was used in each case.

TABLE S2 Nuclear microsatellite locus, motif, allele size range, and annealing temperate in S*chisandra chinensis*.

TABLE S3 Prior distributions for model parameters used in the seven divergence scenarios of *Schisandra chinensis* in DIYABC.

TABLE S4 Selected bioclimatic variables with low correlations (*r* < 0.8) used in ecological niche modeling for *Schisandra chinensis.*

TABLE S5 Haplotypes derived from four chloroplast DNA fragments in *Schisandra chinensis*.

TABLE S6 Haplotypes derived from *PEPC* sequence in *Schisandra chinensis*.

TABLE S7 Genetic diversity and genetic differentiation of eight nuclear microsatellite loci in *Schisandra chinensis*.
