# Supplementary figures and images for: Phylogeography of Schisandra chinensis (Magnoliaceae) Reveal Multiple Refugia With Ample Gene Flow in Northeast China
Source: Front Plant Sci. 2019 Feb 25;10:199. doi: 10.3389/fpls.2019.00199 (PMC6397880; doi:10.3389/fpls.2019.00199)

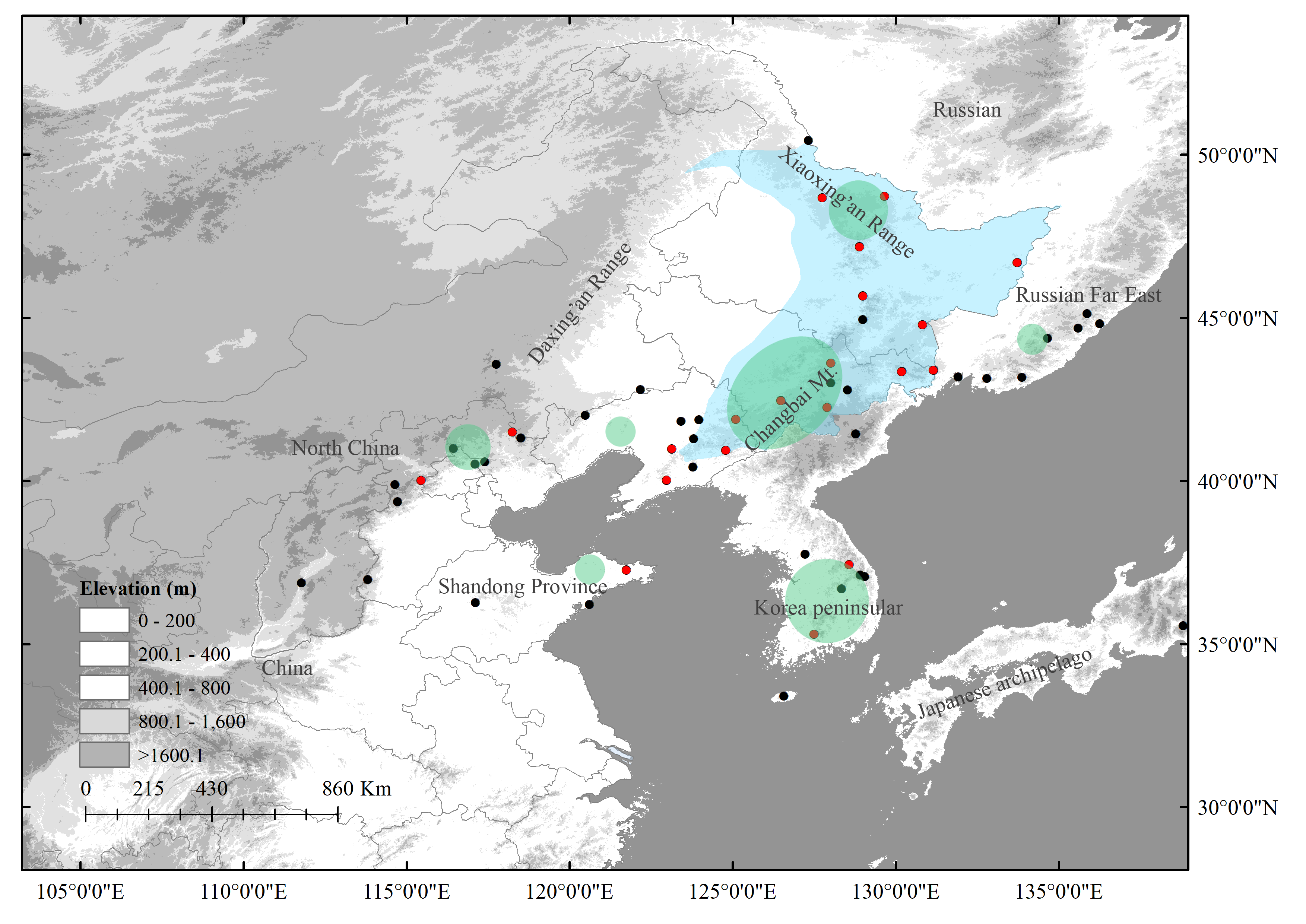

Supplement: FIGURE S1 — Location of the macrorefugia and microrefugia in the temperate conifers and broadleaved mixed forests inferred by Ye et al. (2017). The blue region indicates the range of the mixed forests following Wu (1980). The 35 occurrence records (black dots) obtained from the Global Biodiversity Information Facility database and from the 20 sampled populations (red dots) are shown. [file Image_1.TIF]

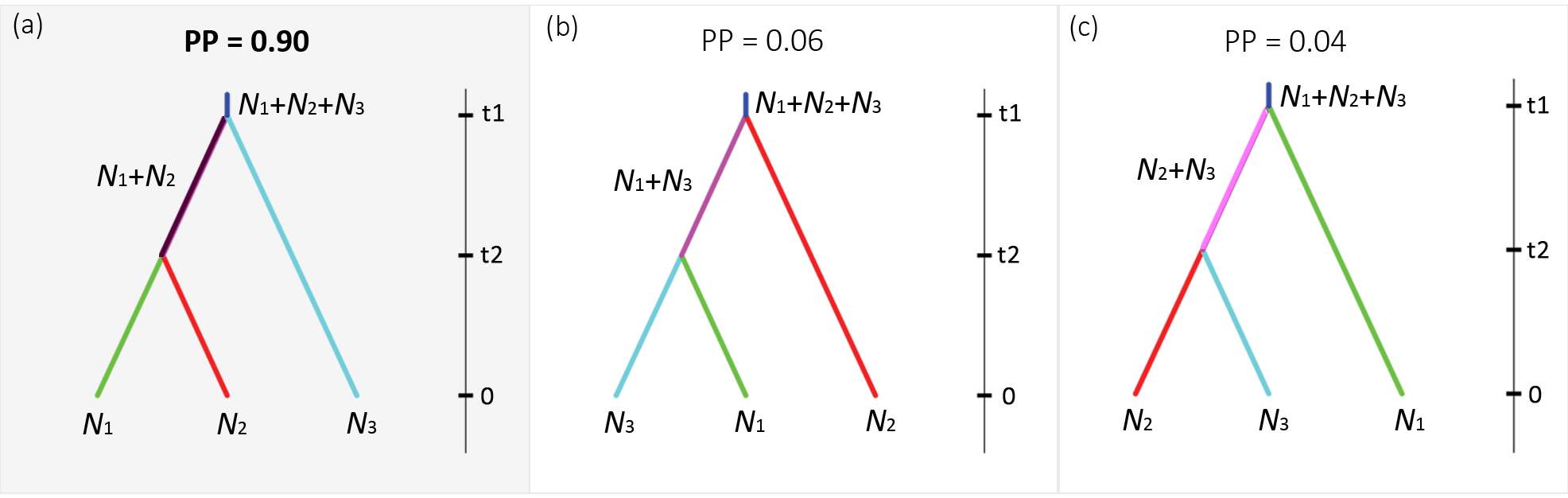

Supplement: FIGURE S2 — Illustration of the three scenarios (a–c) proposed for the divergence history of the three clusters of Schisandra chinensis obtained in DIYABC using eight nuclear microsatellites. N1, N2, and N3 represent the effective population sizes of the east, north, and west genetic clusters inferred by Bayesian clustering. Posterior probability (PP) of the different scenarios is shown. Divergence times for the depicted events are labeled as t1 and t2. [file Image_2.TIF]

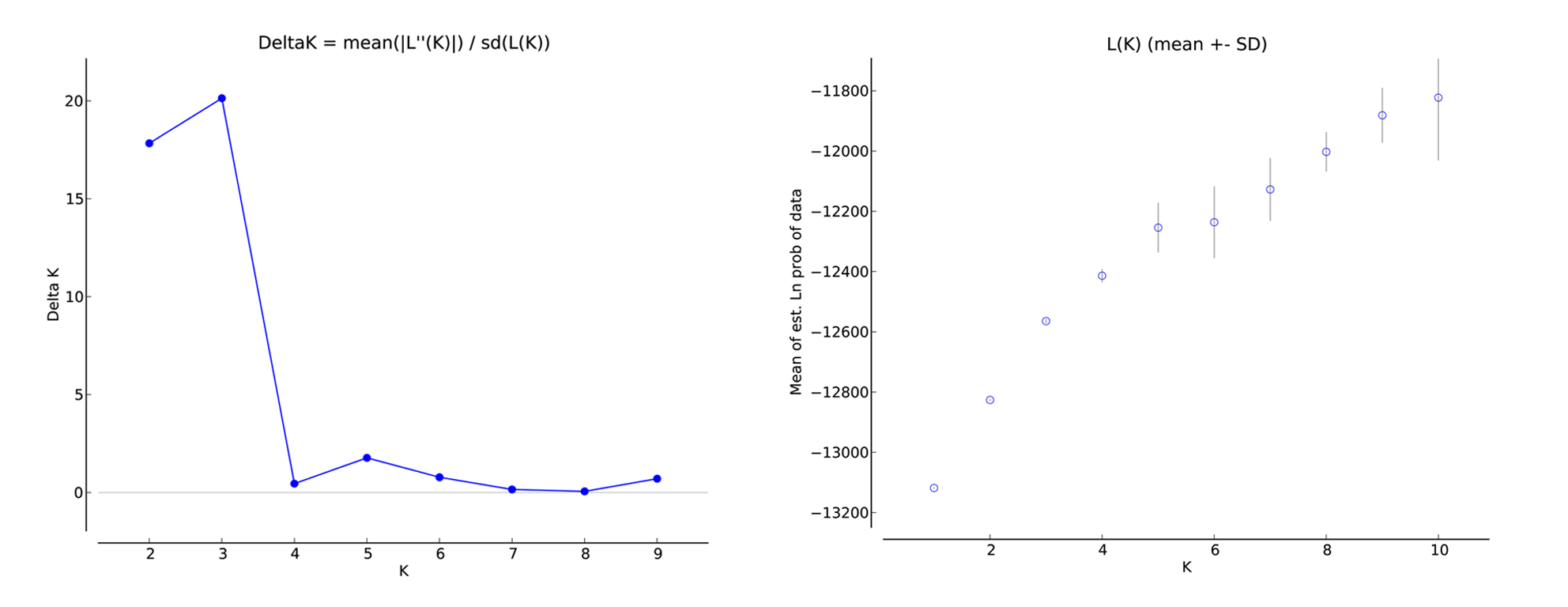

Supplement: FIGURE S3 — ΔK and LnP(D) obtained in the Structure analysis of the 20 Schisandra chinensis populations conducted for predefined group numbers (K = 1–10). The standard deviations of LnP(D) obtained from 10 independent runs for each predefined group size are also shown. [file Image_3.TIF]

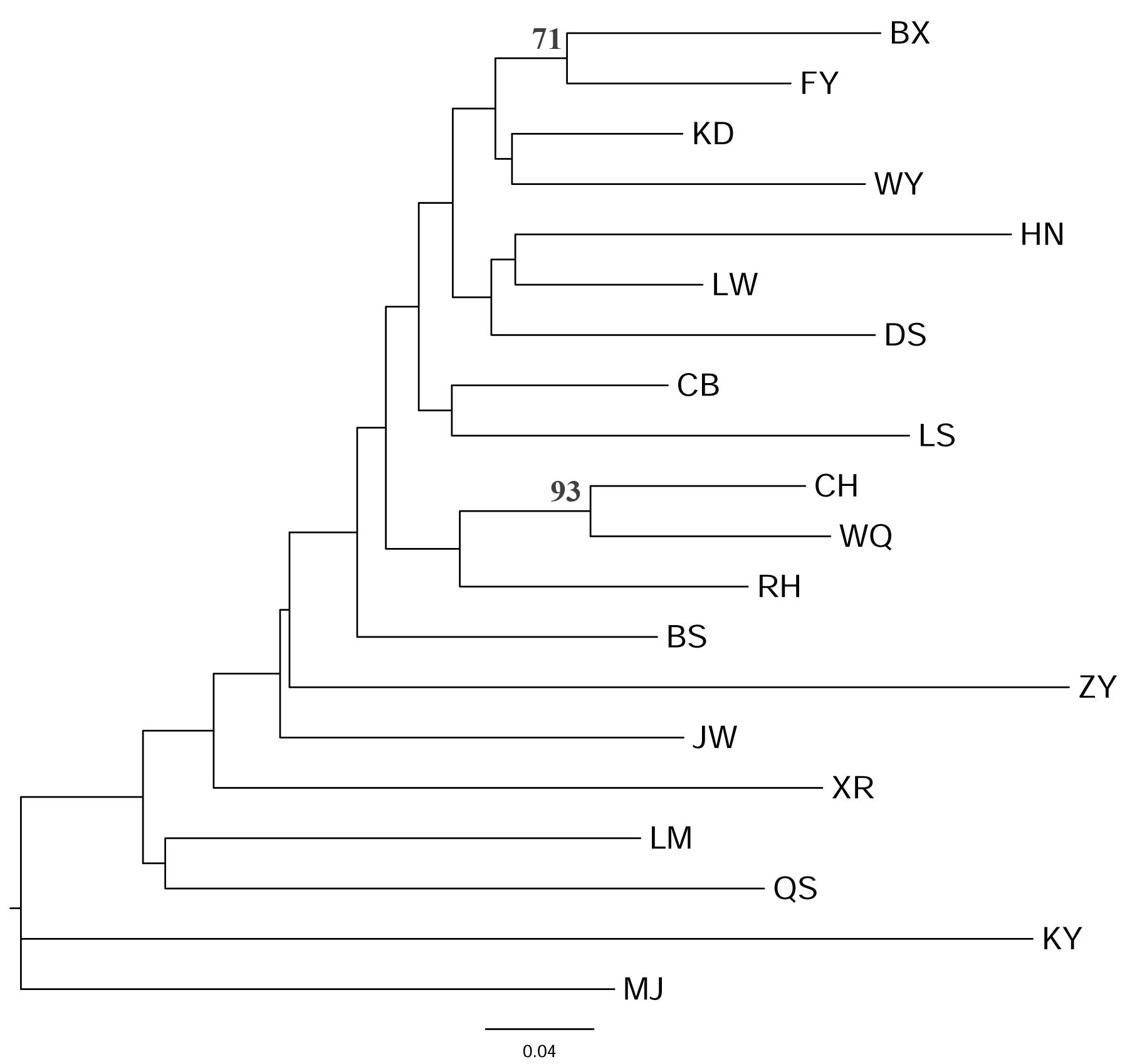

Supplement: FIGURE S4 — Neighbor-joining tree of Schisandra chinensis populations using Nei’s Da as the genetic distance of nSSRs. Bootstrap values above 70% are presented above nodes. [file Image_4.TIF]

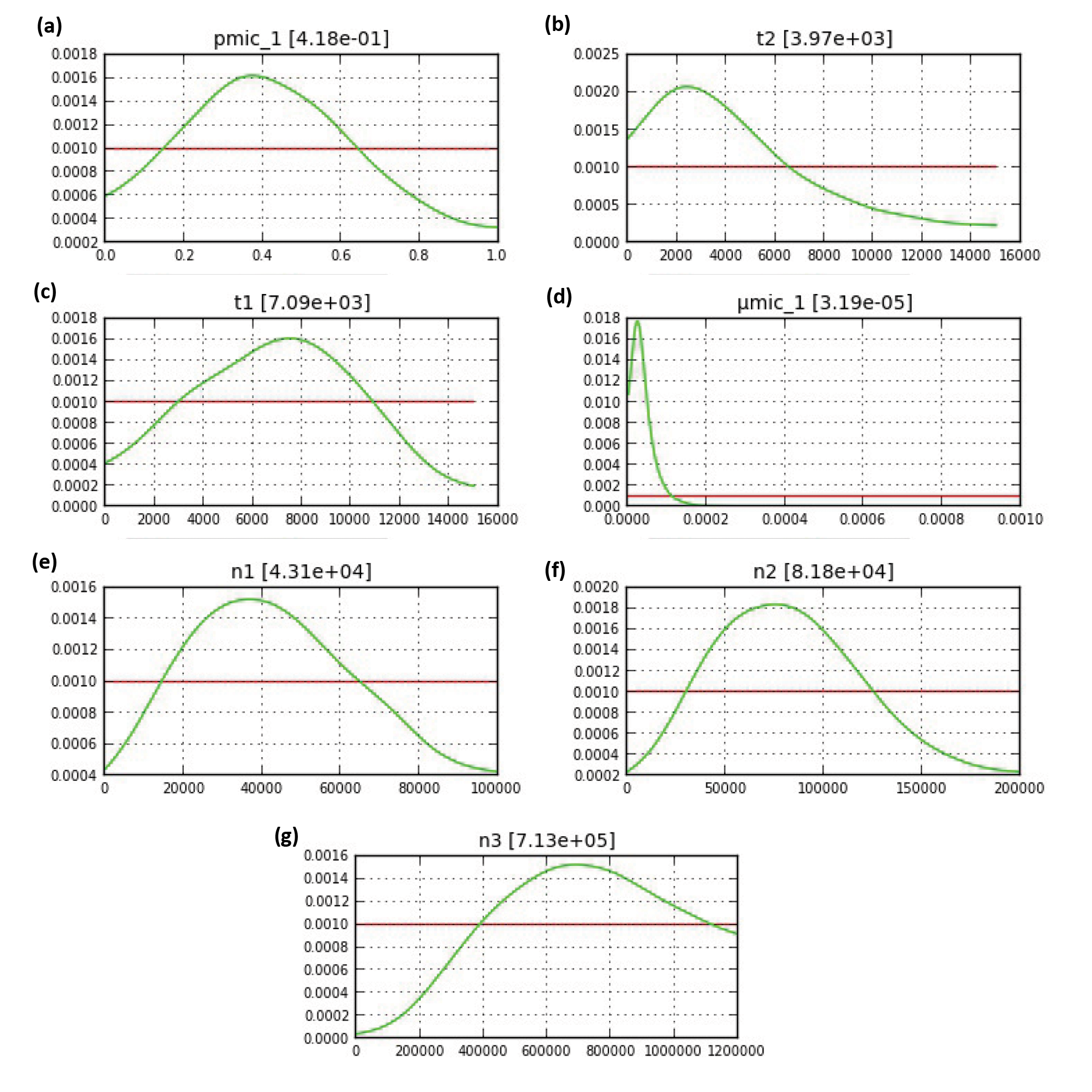

Supplement: FIGURE S5 — Prior (red line) and posterior (green line) distribution of P, the proportion of multiple step mutations in the generalized stepwise model (a), t2, divergence time between west and north Structure clusters (b), t1, divergence time of all three Structure clusters (c), μ, mutation rate (d), and effective population sizes of west (e), north (f), and east (g) Structure clusters. [file Image_5.TIF]
