# Supplementary material for: Phylogeography of Schisandra chinensis (Magnoliaceae) Reveal Multiple Refugia With Ample Gene Flow in Northeast China
Source: Front Plant Sci. 2019 Feb 25;10:199. doi: 10.3389/fpls.2019.00199 (PMC6397880; doi:10.3389/fpls.2019.00199)
Supplement: TABLE S1 — Primers used for chloroplast DNA and nuclear single copy gene sequencings in Schisandra chinensis. An annealing temperature of 54 °C was used in each case. [file Table_1.DOCX]

Supplementary Table S1 Primers used for chloroplast DNA and nuclear single copy gene sequencings in *Schisandra chinensis*. An annealing temperature of 54 °C was used in each case.

| Fragment | Length (bp) | Primer | Primer sequence (5′–3′) | References |
| --- | --- | --- | --- | --- |
| *MatK* | 848 | matK5PSIF | cta tgg ctc caa ttc tgg t | ([Shaw et al., 2005](#_ENREF_2)) |
|  |  | matK5PSIR | ccg cat cag gca cta atc ta |  |
| *ndhA* | 1028 | ndhAx1 | gcy caa tcw att agt tat gaa ata cc | ([Shaw et al., 2007](#_ENREF_3)) |
|  |  | ndhAx2 | ggt tga cgc cam ara ttc ca |  |
| *trnL*–*trnF* | 900 | trnL | cga aat cgg tag acg cta cg | ([Taberlet et al., 1991](#_ENREF_4)) |
|  |  | trnF | att tga act ggt gac acg ag |  |
| *trnS*–*trnG* | 274 | trnS | aga tag gga ttc gaa ccc tcg gt | ([Shaw et al., 2005](#_ENREF_2)) |
|  |  | trnG | ttt tac cac taa act ata ccc gc |  |
| *PEPC* | 809 bp | PPCX4F | act cca cag gat gag atg ag | ([Olson, 2002](#_ENREF_1)) |
|  |  | PPCX5R | gca gcc atc att cta gcc aa |  |

Olson, M.E. (2002). Combining data from DNA sequences and morphology for a phylogeny of Moringaceae (Brassicales). *Systematic Botany* 27**,** 55-73.

Shaw, J., Lickey, E.B., Beck, J.T., Farmer, S.B., Liu, W., Miller, J. et al. (2005). The tortoise and the hare II: relative utility of 21 noncoding chloroplast DNA sequences for phylogenetic analysis. *American Journal of Botany* 92**,** 142-166.

Shaw, J., Lickey, E.B., Schilling, E.E., and Small, R.L. (2007). Comparison of whole chloroplast genome sequences to choose noncoding regions for phylogenetic studies in angiosperms: the tortoise and the hare III. *American Journal of Botany* 94**,** 275-288.

Taberlet, P., Gielly, L., Pautou, G., and Bouvet, J. (1991). Universal primers for amplification of three non-coding regions of chloroplast DNA. *Plant Molecular Biology* 17**,** 1105-1109.
