# Supplementary material for: Phylogeography of Schisandra chinensis (Magnoliaceae) Reveal Multiple Refugia With Ample Gene Flow in Northeast China
Source: Front Plant Sci. 2019 Feb 25;10:199. doi: 10.3389/fpls.2019.00199 (PMC6397880; doi:10.3389/fpls.2019.00199)
Supplement: TABLE S2 — Nuclear microsatellite locus, motif, allele size range, and annealing temperate in Schisandra chinensis. [file Table_2.DOCX]

| Supplementary **Table S2** Nuclear microsatellite locus, motif, allele size range,and annealing temperate in S*chisandra chinensis*. | | | | | |
| --- | --- | --- | --- | --- | --- |
| Locus | Primer sequences (5’-3’) | Repeat motif | Tm (^o^C) | Allele size (bp) | Reference |
| WWZ-WGA36-1 | F: tga gtg ttg caa tac aaa atc ag | (GA)_21_ | 53 | 240-318 | ([Sun et al., 2009](#_ENREF_1)) |
|  | R: gtt tcc agc cca aat ctg aa |  |  |  |  |
| WWZ-WGA36-2 | F: ttc aga ttt ggg ctg gaa ac | (GA)_10_ | 57 | 146-154 | ([Sun et al., 2009](#_ENREF_1)) |
|  | R: tat tct ttc gga gcg aaa cc |  |  |  |  |
| WWZ-C14 | F: agg gtt tgt tca tca gga c | (AG)_24_ | 54 | 175-223 | ([Sun et al., 2009](#_ENREF_1)) |
|  | R: tga ttg tgt tca tct cat gg |  |  |  |  |
| WWZ-C44 | F: gga atc ttt cga aac tca ac | (CT)_15_ | 60 | 136-178 | ([Sun et al., 2009](#_ENREF_1)) |
|  | R: acg aat ctg gtg aga caa ac |  |  |  |  |
| WWZ-WGA18 | F: gct tct agc aag gca ata gca | (AG)_11_ | 63 | 106-130 | ([Sun et al., 2009](#_ENREF_1)) |
|  | R: tcg aat tgg acc gat aca ca |  |  |  |  |
| WWZ-WGA27 | F: gag cac aga cgt ttt cac ca | (AG)_15_ | 60 | 212-278 | ([Sun et al., 2009](#_ENREF_1)) |
|  | R: cga cca cgt ctt cct cta gc |  |  |  |  |
| SS38 | F: acc atc gga atg gaa tca aa | (AC)_6_ | 54 | 236-284 | ([Vernier et al., 2009](#_ENREF_2)) |
|  | R: ttg aga cct cag ccc tga ct |  |  |  |  |
| SS49 | F: tag ttt cca ggc cca tca tc | (TC)_23_ | 57 | 230-306 | ([Vernier et al., 2009](#_ENREF_2)) |
|  | R: ctg gaa tga tgg aag gaa cg | (CA)_18_ |  |  |  |

Sun, Y., Liu, Y.F., and Huang, H.W. (2009). Isolation and characterization of polymorphic microsatellite markers in *Schisandra chinensis* (Turcz.) Baill. (Schisandraceae). *Conservation Genetics Resources* 1**,** 119-121.

Vernier, G., Wang, J., Jennings, L.D., Sun, J., Fischer, A., Song, L. et al. (2009). Isolation and characterization of polymorphic microsatellite loci in a traditional Chinese medicinal plant, *Schisandra sphenanthera*. *Conservation Genetics* 10**,** 615-617.
