# Supplementary material for: Phylogeography of Schisandra chinensis (Magnoliaceae) Reveal Multiple Refugia With Ample Gene Flow in Northeast China
Source: Front Plant Sci. 2019 Feb 25;10:199. doi: 10.3389/fpls.2019.00199 (PMC6397880; doi:10.3389/fpls.2019.00199)
Supplement: TABLE S3 — Prior distributions for model parameters used in the seven divergence scenarios of Schisandra chinensis in DIYABC. [file Table_3.DOCX]

Supplementary **Table S3** Prior distributions for model parameters used in the seven divergence scenarios of *Schisandra chinensis* in DIYABC.

| Parameter | Prior |
| --- | --- |
| *N*_1_ | (10, 100,000) |
| *N*_2_ | (10, 200,000) |
| *N*_3_ | (10, 1200,000) |
| *t_1_* | (10, 150,000) |
| *t_2_* | (10, 150,000) |
| μ | (10^−6^, 10^−3^) |
| *P* | (0.1, 1.0) |

*N*_1_, *N*_2_, N_3_ :the current population size of west cluster, north cluster and east cluster, respectively; *t*_1_: divergence time of three clusters; *t*_2_ divergence time between *N*_1_ and *N*_2_, or *N*_1_ and *N*_3_, or *N*_2_ and *N*_3_; *P*, the proportion of multiple step mutations in the generalized stepwise model. All priors were uniformly distributed. The unit of time is the generation.
