# Supplementary material for: Phylogeography of Schisandra chinensis (Magnoliaceae) Reveal Multiple Refugia With Ample Gene Flow in Northeast China
Source: Front Plant Sci. 2019 Feb 25;10:199. doi: 10.3389/fpls.2019.00199 (PMC6397880; doi:10.3389/fpls.2019.00199)
Supplement: TABLE S4 — Selected bioclimatic variables with low correlations (r < 0.8) used in ecological niche modeling for Schisandra chinensis. [file Table_4.DOCX]

| Supplementary **Table S4** Selected bioclimatic variables with low correlations (*r*  < 0.8) used in ecological niche modelling for *Schisandra chinensis*. | |
| --- | --- |
| Variable | Description |
| BIO1 | Annual mean temperature |
| BIO2 | Mean diurnal range (mean of monthly (max temp - min temp)) |
| BIO3 | Isothermality (BIO2/BIO7*) (× 100) |
| BIO4 | Temperature seasonality (SD × 100) |
| BIO5 | Max temperature of warmest month |
| BIO12 | Annual precipitation |
| BIO15 | Precipitation seasonality (coefficient of variation) |
| *BIO7, annual temperature annual range (BIO5 − BIO6); BIO6, Minimum temperature of the coldest month | |
