# Supplementary material for: Phylogeography of Schisandra chinensis (Magnoliaceae) Reveal Multiple Refugia With Ample Gene Flow in Northeast China
Source: Front Plant Sci. 2019 Feb 25;10:199. doi: 10.3389/fpls.2019.00199 (PMC6397880; doi:10.3389/fpls.2019.00199)
Supplement: TABLE S5 — Haplotypes derived from four chloroplast DNA fragments in Schisandra chinensis. [file Table_5.DOCX]

| Supplementary **Table S5** Haplotypes derived from four chloroplast DNA fragments in *Schisandra chinensis*. | | | | | | | | | | | | | | | |
| --- | --- | --- | --- | --- | --- | --- | --- | --- | --- | --- | --- | --- | --- | --- | --- |
| Haplotype | n | Nucleotide position | | | | | |  |  | |  | |  |  |  |
|  |  | *MatK* | |  | *PEPC* | |  | | | *TrnS-TrnG* | | | |  | *TrnL-TrnF* |
|  |  | 3 | 5 |  | 1 | 1 |  | | | 2 | | 2 | |  | 3 |
|  |  | 1 | 0 |  | 1 | 6 |  | | | 1 | | 9 | |  | 0 |
|  |  | 0 | 3 |  | 3 | 8 |  | | | 1 | | 6 | |  | 2 |
|  |  |  |  |  | 2 | 7 |  | | | 1 | | 3 | |  | 1 |
|  |  | A | A |  | A | C |  | | | T | | A | |  | A |
| H1 | 13 | - | C |  | - | - |  | | | - | | - | |  | - |
| H2 | 105 | - | - |  | - | - |  | | | - | | - | |  | - |
| H3 | 23 | T | - |  | - | - |  | | | - | | G | |  | C |
| H4 | 1 | - | - |  | - | T |  | | | - | | - | |  | - |
| H5 | 1 | - | - |  | G | - |  | | | - | | - | |  | - |
| H6 | 5 | - | - |  | - | - |  | | | C | | - | |  | - |
