# Supplementary material for: Phylogeography of Schisandra chinensis (Magnoliaceae) Reveal Multiple Refugia With Ample Gene Flow in Northeast China
Source: Front Plant Sci. 2019 Feb 25;10:199. doi: 10.3389/fpls.2019.00199 (PMC6397880; doi:10.3389/fpls.2019.00199)
Supplement: TABLE S6 — Haplotypes derived from PEPC sequence in Schisandra chinensis. [file Table_6.DOCX]

| Supplementary **Table S6** Haplotypes derived from *PEPC* sequence in *Schisandra chinensis*. | | | | | | | | | | | | | |
| --- | --- | --- | --- | --- | --- | --- | --- | --- | --- | --- | --- | --- | --- |
| Haplotype | n | Nucleotide position | | | | | | | | | | | |
|  |  | 3 | 3 | 3 | 4 | 4 | 5 | 5 | 6 | 6 | 6 | 7 | 7 |
|  |  | 3 | 6 | 7 | 2 | 4 | 2 | 3 | 3 | 6 | 8 | 6 | 8 |
|  |  | 2 | 5 | 5 | 5 | 3 | 5 | 4 | 7 | 5 | 9 | 5 | 4 |
|  |  | A | T | T | G | G | A | T | T | G | A | A | C |
| H1 | 74 | - | G | - | - | - | - | - | - | - | - | - | - |
| H2 | 50 | - | - | - | - | C | - | - | - | - | T | - | - |
| H3 | 72 | - | G | - | - | - | - | - | - | - | - | G | - |
| H4 | 79 | G | - | - | - | - | - | - | - | - | - | - | - |
| H5 | 40 | G | - | - | - | - | - | - | - | - | - | - | T |
| H6 | 10 | - | - | - | - | - | - | - | - | - | - | - | - |
| H7 | 2 | G | - | C | - | - | - | - | - | - | - | - | - |
| H8 | 1 | - | G | - | - | - | - | C | - | - | - | G | - |
| H9 | 1 | - | G | - | A | - | - | - | - | - | - | G | - |
| H10 | 1 | - | - | - | - | C | - | - | - | A | T | - | - |
| H11 | 3 | - | - | - | - | C | T | - | - | A | T | - | - |
| H12 | 1 | - | G | - | - | - | - | - | C | - | - | - | - |
