# Supplementary material for: Phylogeography of Schisandra chinensis (Magnoliaceae) Reveal Multiple Refugia With Ample Gene Flow in Northeast China
Source: Front Plant Sci. 2019 Feb 25;10:199. doi: 10.3389/fpls.2019.00199 (PMC6397880; doi:10.3389/fpls.2019.00199)
Supplement: TABLE S7 — Genetic diversity and genetic differentiation of eight nuclear microsatellite loci in Schisandra chinensis. [file Table_7.DOCX]

| Supplementary **Table S7** Genetic diversity and genetic differentiation of eight nuclear microsatellite loci in *Schisandra chinensis*. | | | | | |
| --- | --- | --- | --- | --- | --- |
| Locus | *A*_O_ | *H*_O_ | *H*_E_ | *H*_T_ | *F*_ST_ |
| WWZ-WGA36-1 | 30 | 0.84 | 0.84 | 0.93 | 0.13 |
| WWZ-WGA36-2 | 4 | 0.50 | 0.48 | 0.56 | 0.18 |
| WWZ-C14 | 23 | 0.61 | 0.77 | 0.88 | 0.16 |
| WWZ-C44 | 18 | 0.71 | 0.79 | 0.90 | 0.16 |
| SS38 | 20 | 0.74 | 0.75 | 0.75 | 0.15 |
| SS49 | 38 | 0.91 | 0.84 | 0.93 | 0.12 |
| WWZ-WGA18 | 12 | 0.78 | 0.76 | 0.76 | 0.15 |
| WWZ-WGA27 | 22 | 0.77 | 0.78 | 0.88 | 0.15 |
| Mean | 21 | 0.74 | 0.75 | 0.85 | 0.15 |
| *A*_O_, observed allele number; *H*_O_, observed heterozygosity; *H*_E_, expected heterozygosity; *H*_T_, overall gene diversity; *F*_ST_, among population differentiation | | | | | |
